# Loop-mediated isothermal amplification (LAMP) method for detection of genetically modified maize T25

**DOI:** 10.1002/fsn3.68

**Published:** 2013-10-16

**Authors:** Junyi Xu, Qiuyue Zheng, Ling Yu, Ran Liu, Xin Zhao, Gang Wang, Qinghua Wang, Jijuan Cao

**Affiliations:** 1 Food Inspection Center, Liaoning Entry-Exit Inspection and Quarantine BureauDalian, 116001, China; 2 Department of Life Science, Dalian polytechnic UniversityDalian, 116034, China

**Keywords:** GM maize T25, isothermal condition, loop-mediated isothermal amplification, rapid detection

## Abstract

The loop-mediated isothermal amplification (LAMP) assay indicates a potential and valuable means for genetically modified organism (GMO) detection especially for its rapidity, simplicity, and low cost. We developed and evaluated the specificity and sensitivity of the LAMP method for rapid detection of the genetically modified (GM) maize T25. A set of six specific primers was successfully designed to recognize six distinct sequences on the target gene, including a pair of inner primers, a pair of outer primers, and a pair of loop primers. The optimum reaction temperature and time were verified to be 65°C and 45 min, respectively. The detection limit of this LAMP assay was 5 g kg^−1^ GMO component. Comparative experiments showed that the LAMP assay was a simple, rapid, accurate, and specific method for detecting the GM maize T25.

## Introduction

For the increasing world population, conventional breeding of crop varieties cannot meet the demand for food supply. To fulfill the needs, agricultural biotechnology had developed various genetically modified (GM) crops for insect or herbicide resistance, abiotic stress tolerance, or nutritional improvement (Ramessar et al. 2008). Maize is the most widely grown grain crop throughout the world (James 2011). With the increase in various GM maize lines and cultivated areas, the risk of the introduction of GM maize or product into the environment or food chain has become an important issue of bioecology and biosafety concern (Eastham and Sweet 2002). Therefore, it is an important practical significance to detect GM crops from different trading sites.

With the rapid development of the theory and techniques of molecular biology, the new identification technique based on polymerase chain reaction (PCR) technology has been used widely (Lipp et al. 2005, 1999; Chaouachi et al. 2008). Notomi et al. (2000) developed a new thermostat DNA loop-mediated amplification method (loop-mediated isothermal amplification of DNA, LAMP). The principle of LAMP is autocycling strand displacement DNA synthesis in the presence of *Bst* DNA polymerase with high strand displacement activity under isothermal conditions between 60 and 65°C within 60 min. LAMP assays were reported to be highly specific, sensitive, rapid, and cost-effective (Yeh et al. 2006; Han and Ge 2008). In addition, the LAMP assay could be carried out in the regular laboratory, with a water bath or heating block. It is a potential and valuable means for GM food testing.

GM maize T25 has the characteristics of glufosinate herbicide tolerance and is one of the most commonly used strains in processed food and feed (European Commission, Scientific Committee on Plants 2001). To date, research on the application of LAMP in GM maize T25 detection is scare. The objective of the present study was to establish a rapid and accurate LAMP assay for detecting GM maize T25.

## Experimental

### Experimental materials

Nine lines of GM maize were used in this study. GM maize T25 (BF412F), BT176 (BF411F), MON810 (BF413EK), and EVENT98140 (BF427d) were purchased from European Commission, Joint Research Centre, Institute for Reference Materials and Measurements (ERM/IRMM, Geel, Belgium). MIR604 (0607-A2), MON88017 (0406-D), MON89034 (0906-E), MON863 (0406-B), and GA21 (0407-B) were purchased from American Oil Chemists' Society (AOCS, Urbana, IL).

The three lines of GM rice used in this study, GM rice BT63, Kefeng No. 6, Kefeng No. 8, were supplied by Shenyang Academy of Agricultural Sciences (Shenyang, China).

The other seven GM materials were GM soybean A5547-127 (0707-C3, AOCS, Urbana, IL), GM soybean GTS40-3-2 (BF410gk, ERM/IRMM, Geel, Belgium), GM soybean DP305423 (BF426d, ERM/IRMM, Geel, Belgium), GM soybean DP356043 (BF425d, ERM/IRMM, Geel, Belgium), GM soybean MON89788 (0906-B, AOCS, Urbana, IL), GM potato EH92-527-1 (0806-C, AOCS, Urbana, IL), and GM oilseed rape RT73 (0304-B, AOCS, Urbana, IL).

Fresh materials of peas, lentils, green beans, lupins, potatoes, corn, soybeans, chickpeas, mung beans, walnuts, carrots, and wheat were purchased from a supermarket (Carrefour, Dalian, China).

### Genomic DNA extraction

The experimental materials were ground into powder in liquid nitrogen. The genomic DNA was extracted and purified using DNA extraction kit of genetically modified organism (GMO) detection Ver2.0 (No. D9093; TaKaRa, Dalian, China). The extracted genomic DNA was dissolved in 100 μL of Tris-EDTA (TE) solution. The concentration of the purified DNA samples was measured by a ultraviolet–visible spectrometer (ND-1000; NanoDrop, Wilmington, DE), and its quality was evaluated by 1% (w/v) agarose gel electrophoresis.

### LAMP primer design

**Figure 1:**
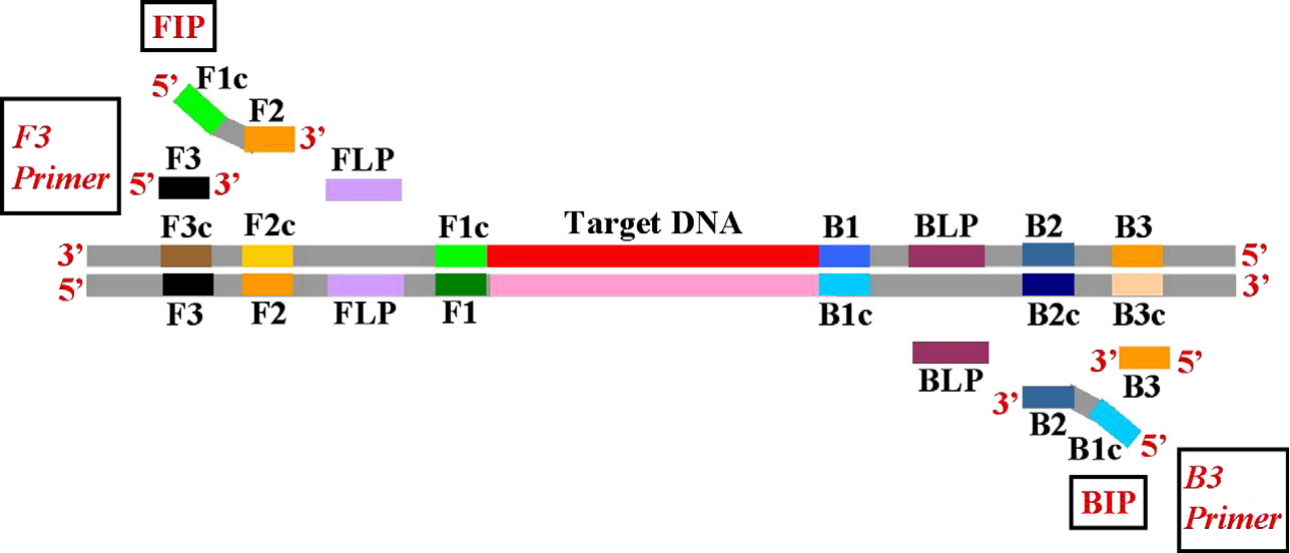
Schematic representation of the set of six specific primers for loop-mediated isothermal amplification (LAMP) assay (F1c and B1c, complementary sequence of F1 and B1, respectively).

Sequence analysis was determined by BLAST program in GenBank database (http://www.ncbi.nlm.nih.gov/BLAST) to distinguish GM T25 strain-specific sequence, and the target fragment covering the maize genome and exogenous sequences connector part. As shown in Figure 1, a set of specific primers were designed for LAMP reaction, including the forward inner primer FIP, back inner primer BIP, two outer primers (F3 and B3), and loop primers (FLP and BLP). Oligonucleotide primers for GM maize T25 were designed using the online software of Primer Explorer version 4 (http://primerexplorer.jp/e) based on the pat gene sequence and the maize genomic DNA (GenBank accession no. AY629235, AX195438, and AX695990). The FIP for GM maize T25 consisted of the complementary sequence of F1 (F1c) and F2: 5′-GGAATCCGAGGAGGTTTCCG-ATCTTCGTCAACATGGTGG-3′. The BIP for GM maize T25 consisted of the complementary sequence of B1 (B1c) and B2: 5′-TTGCCCAGCTATCTGTCACTTT-GCAATGATGGCATTTGTAGG-3′. Primer F3 and B3 for GM maize T25 were 5′-GGAACGACTCAATGACAAGA-3′ and 5′-AGAGGCATCTTCAACGATG-3′. FLP and BLP for GM maize T25 were 5′-TTGGAGTAGACAAGCGTGTC-3′ and 5′-TAGTGGAAAAGGAAGGTGGC-3′, respectively. All primers were synthesized in the concentration of 2 OD by TaKaRa Biotechnology Co. Ltd (Dalian, China).

### LAMP reaction

The LAMP reaction was carried out in a total of 25 μL reaction mixture containing 0.2 μmol/L each outer primer (F3 and B3), 1.6 μmol/L each inner primer (FIP and BIP), 0.8 μmol/L each FLP and BLP, 1.6 mmol/L dNTPs mixture, 1 mol/L betaine (Sigma, Shanghai, China), 6 mmol/L MgSO_4_, 1x ThermoPol buffer (New England Biolabs, Ipswich, MA), 1 μL (8U) *Bst* DNA polymerase (New England Biolabs), and the specified amounts of target genomic DNA. The reaction was incubated at 65°C in a real-time turbidimeter for 45 min, with a termination temperature of 80°C for 5 min.

### Determination of LAMP specificity

Nine lines of GM maize (T25, BT176, EVENT98140, GA21, MON863, MON810, MIR604, MON88017, and MON89034), one non-GM maize, and 21 nonmaize lines (GM rice BT63, Kefeng No. 6, Kefeng No. 8; GM potato EH92-527-1; GM soybean A5547-127, GTS40-3-2, DP305423, DP356043, MON89788; GM oilseed rape GT73; peas, lentils, green beans, lupins, potatoes, soybeans, chickpeas, mung beans, walnuts, carrots, wheat) were used to determine the specificity of the LAMP assay. DNA was extracted as described in the Genomic DNA extraction section. All tests were repeated three times.

### Determination of LAMP sensitivity

To determine the sensitivity of the LAMP assay, the template of GM maize T25 (50 g kg^−1^, 100 ng/μL) was diluted with TE buffer to gradients of 20, 10, 5, and 1 g kg^−1^. Two microliters of each dilution was used as the template for LAMP reaction; ddH_2_O was used in place of the DNA template as a negative control. All tests were repeated three times.

### Real-time PCR reaction

Real-time PCR was also performed for method comparison. The amplification was carried out in a 25 μL reaction system with 10x PCR buffer (with Mg^2+^) 5 μL, dNTPs (10 μmol/L) 2 μL, Taq DNA polymerase (2.5 U) 0.2 μL, forward and reverse primers (10 μmol/L) 0.5 μL, probe (10 μmol/L) 1 μL, template DNA (0.1 and 2 μg) 2 μL, and water up to 25 μL. The real-time PCR reaction conditions were as follows: 95°C for 10 sec, 40 cycles of 95°C for 5 sec, 60°C for 34 sec. The fluorescence signal was collected in each cycle time of annealing. The primer pairs for real-time PCR were 5′-ACAAGCGTGTCGTGCTCCAC-3′ and 5′-GACATGATACTCCTTCCACCG-3′. The probe for real-time PCR was FAM-TCATTGAGTCGTTCCGCCATTGTCG-TAMRA.

## Results

### Specificity of LAMP assay

**Figure 2:**
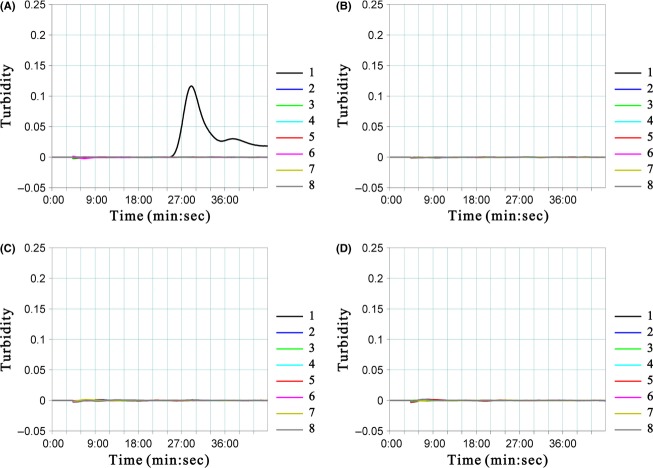
Specificity of LAMP assay. (A) CH1, positive control (GM maize T25); CH2, negative control (ultrapure water); CH3, GM maize MON810; CH4, GM maize MON863; CH5, GM oilseed rape RT73; CH6, GM maize BT176; CH7, GM maize GA21; CH8, GM maize MON89034. (B) CH1, GM potato EH92-527-1; CH2, GM maize MON88017; CH3, GM maize Bt11; CH4, GM soybean MON89788; CH5, GM soybean DP305423; CH6, GM soybean DP356043; CH7, GM soybean GTS40-3-2; CH8, GM maize NK603. (C) CH1, GM rice BT63; CH2, GM rice Kefeng No. 6; CH3, GM rice Kefeng No. 8; CH4, peanuts; CH5, mung bean; CH6, walnut; CH7, carrots; CH8, wheat. (D) CH1, peas; CH2, lentils; CH3, green beans; CH4, lupine; CH5, potatoes; CH6, tomato; CH7, soybean; CH8, chickpeas. LAMP, loop-mediated isothermal amplification; GM, genetically modified.

The LAMP assay run on a real-time turbidimeter successfully detected GM maize T25 in the rest of 30 grain crops (Fig. 2). Time threshold, indicating turbidity values, reach 0.1 and the reaction time is about 29 min. For the 30 non-GM T25 grain crops, no time threshold was obtained, indicating negative results in this study. There were no false-positive or false-negative results in this study and no difference between the LAMP results detected by the naked eye or turbidity in a real-time turbidimeter. The GM T25 maize was simultaneously detected by real-time PCR using the same DNA templates. The results of real-time PCR were consistent with the results of LAMP.

### Sensitivity of LAMP assay

LAMP and RT-PCR assays were carried out using diluted T25 genomic DNA templates (100, 50, 10, 5, 1, and 0.5 g kg^−1^) to compare the sensitivities of LAMP and RT-PCR. When the dilution reached a DNA concentration of 1 g kg^−1^, the LAMP method did not successfully amplify the target DNA products. As for the RT-PCR method, the detection limit was possible to reach 0.5 g kg^−1^; the results have been validated with 20-time repeatability.

**Figure 3:**
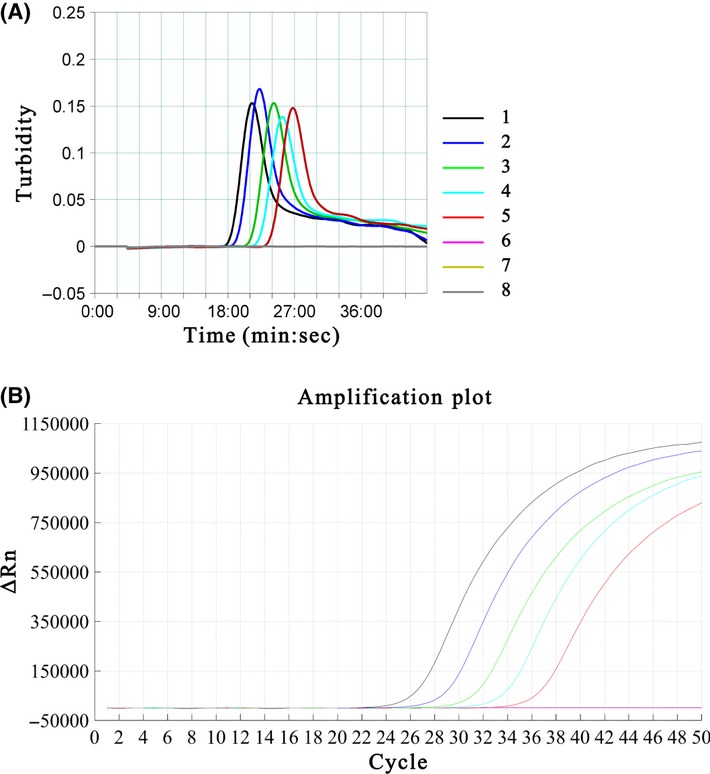
Compare the sensitivity of LAMP assay with real-time PCR. (A) CH1, 1000 g kg^−1^ GM maize T25; CH2, 100 g kg^−1^ GM maize T25; CH3, 50 g kg^−1^ GM maize T25; CH4, 10 g kg^−1^GM maize T25; CH5, 5 g kg^−1^ GM maize T25; CH6, 1 g kg^−1^ GM maize T25; CH7, 0.5 g kg^−1^ GM maize T25; CH8, negative control (ultrapure water). (B) 1, 100 g kg^−1^ GM maize T25; 2, 10 g kg^−1^ GM maize T25; 3, 5 g kg^−1^ GM maize T25; 4, 1 g kg^−1^ GM maize T25; 5, 0.5 g kg^−1^ GM maize T25. LAMP, loop-mediated isothermal amplification; PCR, polymerase chain reaction; GM, genetically modified.

The LAMP reaction was positive for the sample containing 5 g kg^−1^ GM maize T25 or more (Fig. 3A), indicating that the sensitivity of the LAMP assay was 5 g kg^−1^. The sensitivity of real-time PCR was 0.5 g kg^−1^ (Fig. 3B), up to 10-fold more sensitive than the LAMP assay.

**Figure 4:**
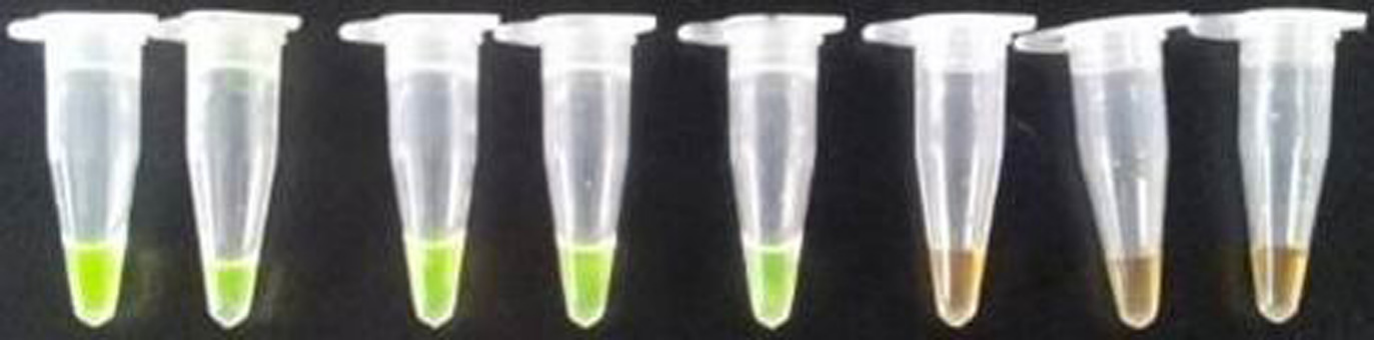
Visual inspection of the LAMP products following the addition of SYBR Green I. 1, 1000 g kg^−1^ GM maize T25; 2, 100 g kg^−1^ GM maize T25; 3, 50 g kg^−1^ GM maize T25; 4, 10 g kg^−1^ GM maize T25; 5, 5 g kg^−1^ GM maize T25; 6, 1 g kg^−1^ GM maize T25; 7, 0.5 g kg^−1^ GM maize T25; 8, negative control (ultrapure water). LAMP, loop-mediated isothermal amplification; GM, genetically modified.

In a real-time turbidimeter, the time threshold values were between 21 and 27 min for templates ranging from 1000 to 5 g kg^−1^ concentration of GM maize T25. The LAMP products turned green after the addition of SYBR Green I. Thus, the sensitivity of the LAMP methods can simply be judged by the naked eye (Fig. 4). Forty-five minutes of incubation was sufficient to allow the minimal amount of template DNA to amplify and to be detected by the naked eye. Sensitivity detected by naked eye or turbidity identical matched with each other.

### Stability of LAMP assay

**Figure 5:**
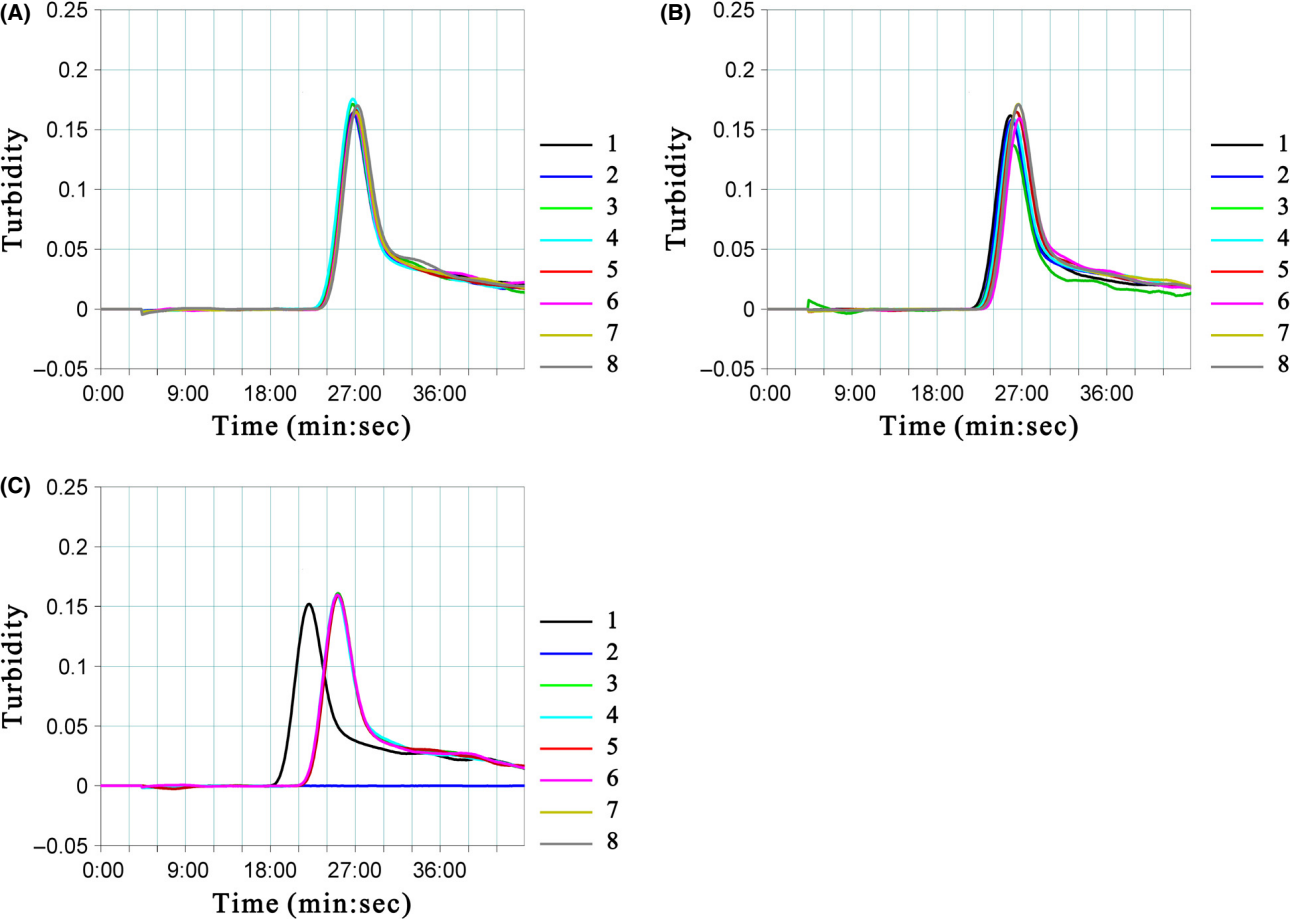
Stability of LAMP assay. (A) CH1–8, 5 g kg^−1^ GM maize T25. (B) CH1–8, 5 g kg^−1^ GM maize T25. (C) CH1, positive control (1000 g kg^−1^ GM maize T25); CH2, negative control (ultrapure water); CH3–6, 5 g kg^−1^ GM maize T25. LAMP, loop-mediated isothermal amplification; GM, genetically modified.

The consistency of the results from the LAMP assay was detected using 5 g kg^−1^ T25 genomic DNA templates for 20-time repeatability. The time threshold values were between 24 and 27 min for 20 parallel templates (Fig. 5), showing a good stability in concentration of 5 g kg^−1^.

## Discussion

As a novel nucleic acid amplification method, LAMP has already been applied for the detection of several GM crops and their components. Fukuta et al. (2004) established a LAMP method for the detection of CaMV35S promoter in transgenic tomato and its products: 5–50 g kg^−1^ content of transgenic component could be detected. Lan et al. (2008) designed four specific primer targets for six regions of CP4-epsps synthase gene and established a LAMP detection method for GM soybean CP4-epsps gene. Lee et al. (2009) detected CaMV35S promoter, nopaline synthase gene promoter, and terminator in transgenic rapeseed by LAMP. The transgenic component can be detected from a single copy template.

Currently, methods for the detection of GM corn are mainly TaqMan real-time PCR and conventional PCR methods. Due to the shortage of complicated operation, environment pollution, and low sensitivity, the conventional PCR method has been gradually replaced by the real-time PCR assay. The real-time PCR assay has many advantages over conventional PCR, including rapidity, a low contamination rate, a higher sensitivity, and easy standardization, but it requires fluorogenic primers and probes as well as expensive detection equipment. The high costs of the instruments required to perform real-time assays restrict their use to laboratories with good financial resources.

LAMP assays are highly specific, sensitive, rapid, and cost-effective. In addition, the LAMP assay is carried out under isothermal condition; therefore, no special apparatus is needed. A regular laboratory water bath or heating block is good enough (Wang et al. 2008). The LAMP assay is able to synthesize large amounts of gene products. Accordingly, a large amount of by-product, pyrophosphate ion, is produced, yielding a white precipitate of magnesium pyrophosphate in the reaction mixture. Turbidity would increase in correlation with DNA yield. As an increase in the turbidity of the reaction mixture according to the production of precipitate correlates with the amount of DNA synthesized, real-time monitoring of the LAMP reaction was achieved by real-time measurement of turbidity (Mori et al. 2004). The results could also be inspected by the naked eye. The LAMP reaction mixture turned green after the addition of SYBR Green I, whereas a solution with no amplicons retained the original orange color of SYBR Green I. Therefore, this method facilitates the application of LAMP, especially in field laboratory settings.
